# Supplementary material for: Dual matter-wave inertial sensors in weightlessness
Source: Nat Commun. 2016 Dec 12;7:13786. doi: 10.1038/ncomms13786 (PMC5159825; doi:10.1038/ncomms13786)
Supplement: Supplementary Information — Supplementary Figures 1-2, Supplementary Notes 1-2 and Supplementary References. [file ncomms13786-s1.pdf]

Supplementary Information

B. Barrett,[1,*] L. Antoni-Micollier,[1] L. Chichet,[1] B. Battelier,[1] T. Lévèque,[2] A. Landragin,[3] and P. Bouyer[1]

[1]*LP2N, IOGS, CNRS and Université de Bordeaux, rue François Mitterrand, 33400 Talence, France*
[2]*CNES, 18 avenue Edouard Belin, 31400 Toulouse, France*
[3]*LNE-SYRTE, Observatoire de Paris, PSL Research University, CNRS, Sorbonne Universités, UPMC Univ. Paris 06, 61 avenue de l'Observatoire, 75014 Paris, France*

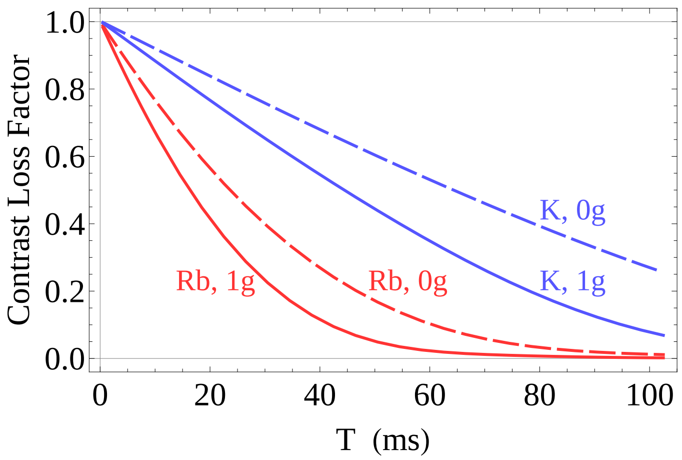

Supplementary Figure 1. Relative contrast loss due to vibrations as a function of interrogation time T. The curves are based on measured experimental parameters for the ^{87}Rb (red curves) and ^{39}K (blue curves) interferometers while operating in $1g$ (solid curves) and $0g$ (dashed curves). Model parameters: standard deviation of vibration noise during $1g$ ($\sigma_a^{\rm vib} \simeq 0.055\,g$) and during $0g$ ($0.038\,g$); atomic sample temperatures $\mathcal{T}_{\rm Rb} \simeq 4$ μK, $\mathcal{T}_{\rm K} \simeq 18$ μK; π-pulse durations $\tau_{\rm Rb}^{\pi} \simeq 15$ μs, $\tau_{\rm K}^{\pi} \simeq 9$ μs; selected velocities $v_{\rm Rb}^{\rm sel} \simeq 3$ cm s^{-1}, $v_{\rm K}^{\rm sel} \simeq 8$ cm s^{-1}.

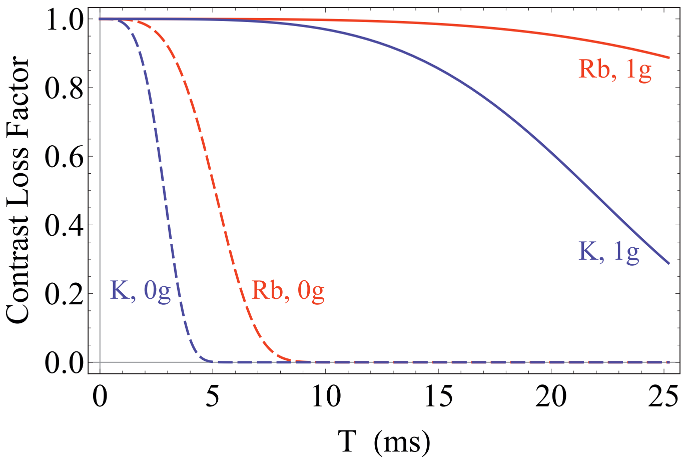

Supplementary Figure 2. Rotation-induced contrast loss factor as a function of T for ^{87}Rb (red curves) and ^{39}K (blue curves), during steady flight (solid curves) and parabolic flight (dashed curves). These results were computed from Supplementary Equation (18) using the measured sample temperatures $\mathcal{T}_{\rm Rb} \simeq 4$ μK and $\mathcal{T}_{\rm K} \simeq 18$ μK, and the mean rotation rates displayed in Table 3 of the main text.

* brynle.barrett@institutoptique.fr

SUPPLEMENTARY NOTE 1: CONTRAST LOSS DUE TO MIRROR VIBRATIONS

Here we provide a description of the model we developed for interferometer contrast loss due to mirror vibrations. Vibrations of the inertial reference mirror cause a time-dependent Doppler shift $\delta_j^{\rm vib}(t)$ of the Raman beam frequency in the rest frame of the atoms. This frequency shift is given by the integral of the acceleration noise $\boldsymbol{a}^{\rm vib}(t)$ experienced by the retro-reflection mirror

$$\delta_j^{\rm vib}(t) = \boldsymbol{k}_j^{\rm eff} \cdot \int_{t_0}^{t} \boldsymbol{a}^{\rm vib}(t')\mathrm{d}t', \tag{1}$$

where t_0 represents the time of the first Raman pulse. During the interferometer sequence, $\delta_j^{\rm vib}(t)$ must be well within the bandwidth of each Raman pulse, as determined by the effective Rabi frequency $\Omega_j^{\rm eff}$ for atom j, in order to address the same velocity class of atoms. More specifically, we require

$$\left|\delta_j(\boldsymbol{v},t) - \delta_j^{\rm vib}(t)\right| \ll \Omega_j^{\rm eff} \tag{2}$$

for $t - t_0 = 0, T_j$, and $2T_j$, where δ_j is the two-photon Raman detuning described by

$$\delta_j(\boldsymbol{v},t) = \delta_j^{\rm las}(t) - \delta_j^{\rm res}(\boldsymbol{v},t), \tag{3}$$

$$\delta_j^{\rm las}(t) = \delta_j^{\rm rf} + \alpha_j t, \tag{4}$$

$$\delta_j^{\rm res}(\boldsymbol{v},t) = \delta_j^0 + \boldsymbol{k}_j^{\rm eff} \cdot \big(\boldsymbol{v} + \boldsymbol{a}t\big). \tag{5}$$

Here, $\delta_j^{\rm las}$ is the frequency difference between Raman lasers and $\delta_j^{\rm res}$ is the two-photon resonance frequency which contains three main contributions: (i) a constant shift $\delta_j^0 = \omega_j^{\rm HF} + \omega_j^{\rm rec}$, which includes the hyperfine splitting between ground states $\omega_j^{\rm HF}$ and the two-photon recoil frequency $\omega_j^{\rm rec} = \hbar(k_j^{\rm eff})^2/2M_j$, (ii) the velocity-dependent Doppler shift $\boldsymbol{k}_j^{\rm eff} \cdot \boldsymbol{v}$, and (iii) a time-dependent shift due to an acceleration of the atoms $\boldsymbol{k}_j^{\rm eff} \cdot \boldsymbol{a}t$. The Raman frequency difference consists of a radio frequency (rf) offset to cancel the first two components $\delta_j^{\rm rf} \simeq \delta_j^0 + \boldsymbol{k}_j^{\rm eff} \cdot \boldsymbol{v}$, plus a frequency chirp $\alpha_j \simeq |\boldsymbol{k}_j^{\rm eff} \cdot \boldsymbol{a}|$ used to cancel the projection of atomic acceleration along the Raman beams. Using these simplifications, condition (2) can be reduced to

$$\left|\boldsymbol{k}_j^{\rm eff} \cdot \left(\boldsymbol{v} - \boldsymbol{v}_j^{\rm sel} + \int_{t_0}^{t} \boldsymbol{a}^{\rm vib}(t')\mathrm{d}t'\right)\right| \ll \Omega_j^{\rm eff}, \tag{6}$$

where $\boldsymbol{v}_j^{\rm sel} = (\delta_j^{\rm rf} - \delta_j^0)\epsilon_{\boldsymbol{k}}/k_j^{\rm eff}$ is the selected velocity class determined by the rf component of the Raman frequency. If condition (6) is not satisfied during each pulse, there is a loss of interference contrast due to a diminished excitation efficiency for the same velocity class of atoms. As a function of T, the degree to which contrast is lost will depend on the vibration spectrum defined by $\boldsymbol{a}^{\rm vib}(t)$. Since we are concerned with only the average behavior of the system, we treat the vibrations as random white noise with a standard deviation $\sigma_a^{\rm vib}$. Intuitively, the loss of contrast becomes significant when $\sigma_a^{\rm vib} \gtrsim \Omega_j^{\rm eff}/k_j^{\rm eff}T_j$.

To obtain a quantitative model for the contrast loss of the interferometer, we use a semi-classical description for the interaction with each Raman pulse. We then propagate an atomic wavepacket through the standard Mach-Zehnder interferometer sequence using an operator approach. We consider an atomic wavefunction in the momentum space representation given by

$$|\Psi(\boldsymbol{p},t)\rangle = \Phi_0(\boldsymbol{p}) \sum_{F=1}^{2} c_F(\boldsymbol{p},t)\,|F,\boldsymbol{p}\rangle\,, \tag{7}$$

where $\Phi_0(\boldsymbol{p}) = \frac{1}{(\sqrt{\pi}\sigma_p)^{3/2}} e^{-\frac{1}{2}(\boldsymbol{p}/\sigma_p)^2}$ is the initial Gaussian wavepacket probability amplitude, with e^{-1} width $\sigma_p = \sqrt{2Mk_B\mathcal{T}}$ corresponding to an ensemble temperature $\mathcal{T}$. At this point, we drop the subscript j to avoid a cumbersome notation. The time-dependent coefficients c_F contain all of the dynamics concerning the wavepackets during the sequence. One can write the evolution of these coefficients during a Raman pulse of duration τ as

$$\begin{pmatrix} c_1(\boldsymbol{p}, t_0+\tau) \\ c_2(\boldsymbol{p}+\hbar\boldsymbol{k}^{\rm eff}, t_0+\tau) \end{pmatrix} = \hat{U}_{\rm int}(\Theta,\delta,\varphi) \begin{pmatrix} c_1(\boldsymbol{p}, t_0) \\ c_2(\boldsymbol{p}+\hbar\boldsymbol{k}^{\rm eff}, t_0) \end{pmatrix}. \tag{8}$$

where the interaction with the Raman beams is described by the following operator [1, 2]

$$\hat{U}_{\rm int}(\Theta,\delta,\varphi) = \begin{pmatrix} \big(\cos\frac{\Theta}{2} - i\frac{\delta\tau}{2}\mathrm{sinc}\frac{\Theta}{2}\big)e^{-i\delta\tau/2} & -i\frac{\Omega^{\rm eff}\tau}{2}\mathrm{sinc}\frac{\Theta}{2}e^{-i\delta\tau/2}e^{-i\varphi} \\ -i\frac{\Omega^{\rm eff}\tau}{2}\mathrm{sinc}\frac{\Theta}{2}e^{i\delta\tau/2}e^{i\varphi} & \big(\cos\frac{\Theta}{2} + i\frac{\delta\tau}{2}\mathrm{sinc}\frac{\Theta}{2}\big)e^{i\delta\tau/2} \end{pmatrix}. \tag{9}$$

Here, φ is the phase difference between Raman lasers during the pulse and $\Theta(\boldsymbol{p}) = \left({\Omega^{\rm eff}}^2 + \delta^2\right)^{1/2}\tau$ is the effective pulse area corresponding to pulse length τ, Rabi frequency $\Omega^{\rm eff}$, and velocity-dependent Raman detuning $\delta(\boldsymbol{v}) = \boldsymbol{k}^{\rm eff} \cdot (\boldsymbol{v}^{\rm sel} - \boldsymbol{v})$. Operating on an atom initially in state $|1,\boldsymbol{p}\rangle$ with pulse area $\Theta(\boldsymbol{p}) = \pi/2$ corresponds to an atomic beam splitter that diffracts the atom into a 50/50 superposition of momentum states $|1,\boldsymbol{p}\rangle$ and $\left|2,\boldsymbol{p}+\hbar\boldsymbol{k}^{\rm eff}\right\rangle$. Similarly, $\Theta = \pi$ corresponds to an atomic mirror—inverting the population between these two states. To construct a Mach-Zehnder interferometer (*i.e.* a $\pi/2 - \pi - \pi/2$ pulse sequence), we evaluate the following operator product

$$\hat{U}_{\rm MZ}(T,\delta) = \hat{U}_{\rm int}(\pi/2,\delta,\varphi_3)\hat{U}_{\rm free}(T)\hat{U}_{\rm int}(\pi,\delta,\varphi_2)\hat{U}_{\rm free}(T)\hat{U}_{\rm int}(\pi/2,\delta,\varphi_1), \tag{10}$$

where $\hat{U}_{\rm free}(t)$ is a unitary operator that evolves the wavefunction in free space for a time t

$$\hat{U}_{\rm free}(t) = \begin{pmatrix} e^{-iE_1 t/\hbar} & 0 \\ 0 & e^{-iE_2 t/\hbar} \end{pmatrix}, \tag{11}$$

with energies $E_1 = \boldsymbol{p}^2/2M$ and $E_2 = \boldsymbol{p}^2/2M + \hbar\omega^{\rm HF}$. The wavefunction at the output of the interferometer is then given by $|\Psi(\boldsymbol{p},2T)\rangle = \hat{U}_{\rm MZ}\,|\Psi(\boldsymbol{p},0)\rangle$, and the probability of finding the atom in either output port of the interferometer is

$$P_F = \int {\rm d}\boldsymbol{p}\, \left|\langle F,\boldsymbol{p}\,|\Psi(\boldsymbol{p},2T)\rangle\right|^2 \equiv \langle P_F\rangle \pm \frac{C}{2}\cos(\varphi_1 - 2\varphi_2 + \varphi_3), \tag{12}$$

where $\langle P_F\rangle$ is a constant probability offset for state $|F=1,2\rangle$, C is the fringe contrast, and the sign corresponds to a given output port $|F\rangle$.

We simulate the contrast loss due to vibrations as a time-varying shift of the Raman detuning by replacing δ in Supplementary Equation (10) with $\delta + \delta^{\rm vib}_{\rm rms}(t)$, where

$$\delta^{\rm vib}_{\rm rms}(t) \simeq k^{\rm eff}\sigma^{\rm vib}_a(t - t_0) \tag{13}$$

is the root-mean-square Doppler shift from vibrations at frequencies $\lesssim 1/T$. The contrast is extracted numerically by varying the total laser phase over 2π and measuring the maximum excursion of the population in either state. Supplementary Figure 1 shows estimates of the relative contrast loss from mirror vibrations based on measured experimental parameters for our ^{87}Rb and ^{39}K interferometers during the different phases of flight onboard the Zero-G aircraft. Clearly, the present level of mirror vibrations has a detrimental effect on the fringe contrast after only a few tens of milliseconds. We note that the potassium interferometer is able to retain contrast for longer interrogation times due to the use of a smaller π-pulse duration compared to rubidium. This results in a broader pulse bandwidth $\Omega^{\rm eff}$ and thus a lower sensitivity to vibrations.

During the analysis of the interferometer fringes shown in Figure 3 of the main text, we test condition (6) using measurements of the mirror vibrations. Points that do not satisfy this condition corrupt the fringes and are therefore removed.

SUPPLEMENTARY NOTE 2: CONTRAST LOSS DUE TO ROTATIONS

In this note, we describe the model used for interferometer contrast loss due to rotations of the Raman beams. Rotations of the Raman wavevector cause a separation of atomic trajectories at the final $\pi/2$ pulse. This "open interferometer" effect can be quantified by an overlap integral between the wavepackets at either output port of the interferometer [3, 4]. Under reasonable approximations, this overlap integral can be written as [4]

$$C \propto \left| \int {\rm d}^3 r \exp\left(i\Delta\boldsymbol{\chi}\cdot\boldsymbol{r}/\hbar\right) |\psi_{\rm c}(\boldsymbol{r},2T)|^2 \right|, \tag{14}$$

where $|\psi_{\rm c}(\boldsymbol{r},t)\rangle$ is the spatial atomic wavefunction for the center-of-mass momentum state $\left|\boldsymbol{p}+\frac{1}{2}\hbar\boldsymbol{k}^{\rm eff}\right\rangle$, and $\Delta\boldsymbol{\chi}$ is the effective displacement momentum between the wavepackets that traveled along the upper and lower interferometer pathways at time $t = 2T$ as given by

$$\Delta\boldsymbol{\chi} = M\left(\Delta\boldsymbol{v} - \frac{\Delta\boldsymbol{r}}{2T}\right). \tag{15}$$

To obtain this displacement momentum vector, we have evaluated the classical trajectories of the interferometers by solving the Euler-Lagrange equations using the Lagrangian

$$L(\boldsymbol{r},\boldsymbol{v}) = \frac{1}{2}M\left(\boldsymbol{v} + \boldsymbol{\Omega}\times\boldsymbol{r}\right)^2 + M\boldsymbol{a}\cdot\boldsymbol{r}, \tag{16}$$

which describes the motion of non-interacting particles with mass M, undergoing a constant rotation $\boldsymbol{\Omega} = \{\Omega_x, \Omega_y, \Omega_z\}$ and a constant acceleration $\boldsymbol{a}$, where the vectors $\boldsymbol{r}$ and $\boldsymbol{v} = \dot{\boldsymbol{r}}$ are defined in the rotating frame. For interferometry beams aligned along the z-axis (*i.e.* $\boldsymbol{k}^{\rm eff} = k^{\rm eff}\,\boldsymbol{\epsilon}_z$), the displacement vector can be shown to be (up to second order in T and $\boldsymbol{\Omega}$)

$$\Delta\boldsymbol{\chi} = \frac{\hbar k^{\rm eff}}{2}\begin{pmatrix} 2\Omega_y T + 3\Omega_x\Omega_z T^2 \\ -2\Omega_x T + 3\Omega_y\Omega_z T^2 \\ -3(\Omega_x^2 + \Omega_y^2)T^2 \end{pmatrix}. \tag{17}$$

This quantity is independent of the acceleration $\boldsymbol{a}$, initial velocity $\boldsymbol{v}_0$ and initial position $\boldsymbol{r}_0$ of the particle, which implies that the loss of contrast due to rotations can be easily separated from that due to mirror vibrations, for instance. To model the loss of contrast in our experiment, we evaluate the overlap integral (14) using a Gaussian wavepacket corresponding to a temperature $\mathcal{T}$, with zero mean velocity. The resulting contrast loss is then described by

$$C \propto \exp\left[-\left(\frac{k^{\rm eff}\sigma_r(2T)T}{2}\right)^2(\Omega_x^2 + \Omega_y^2)\left(1 + \frac{9}{4}|\boldsymbol{\Omega}|^2 T^2\right)\right], \tag{18}$$

where $\sigma_r(t) = \sqrt{(\hbar/\sigma_p)^2 + (\sigma_p t/M)^2}$ is the e^{-1} radius of the Gaussian wavepacket in position space, and $\sigma_p = \sqrt{2k_B M\mathcal{T}}$ is the corresponding width in momentum space.

Supplementary Figure 2 displays the relative loss of contrast due to the nominal rotations of the Zero-G aircraft, as estimated from Supplementary Equation (18) for both rubidium and potassium interferometers. Due to the larger temperature of the potassium sample, the contrast loss is stronger than for rubidium. During steady flight, we estimate that the ^{39}K interferometer contrast reduces by a factor of 2 by $T \sim 20$ ms, while in parabolic flight the same loss occurs by only $T \sim 3$ ms due to the increased rotation rate Ω_y. This represents the most significant limitation for the interferometers in the aircraft.

SUPPLEMENTARY REFERENCES

[1] Kathryn Moler, David S. Weiss, Mark Kasevich, and Steven Chu, "Theoretical analysis of velocity-selective Raman transitions," Phys. Rev. A **45**, 342–348 (1992).

[2] J. M. Hogan, D. M. S. Johnson, and M. A. Kasevich, "Light-pulse atom interferometry," in *Proceedings of the International School of Physics "Enrico Fermi"*, **168**, 411–447 (2009), E. Arimondo, W. Ertmer, W. P. Schleich, and E. M. Rasel (eds.) (IOS, Amsterdam; SIF, Bologna).

[3] Shau-Yu Lan, Pei-Chen Kuan, Brian Estey, Philipp Haslinger, and H. Müller, "Influence of the Coriolis Force in Atom Interferometry," Phys. Rev. Lett. **108**, 090402 (2012).

[4] A. Roura, W. Zeller, and W. P. Schleich, "Overcoming loss of contrast in atom interferometry due to gravity gradients," New J. Phys. **16**, 123012 (2014).
